# Printability and Microstructure of Selective Laser Melting of WC/Co/Cr Powder

**DOI:** 10.3390/ma12152397

**Published:** 2019-07-27

**Authors:** Sabina Luisa Campanelli, Nicola Contuzzi, Paolo Posa, Andrea Angelastro

**Affiliations:** Department of Mechanics, Mathematics and Management, Politecnico di Bari, Viale Japigia 182, 70126 Bari, Italy

**Keywords:** selective laser melting, additive manufacturing, metal powders, tungsten carbide, printability, density, microstructure

## Abstract

The selective laser melting process is a growing technology for the manufacture of parts with very complex geometry. However, not all materials are suitable for this process, involving rapid localized melting and solidification. Tungsten has difficulties due to the high melting temperature. This study focuses on the possibility of processing a WC/Co/Cr composite powder using selective laser melting. Samples were fabricated and characterized in terms of density, defects, microstructure and hardness. Tests were conducted with hatch spacing of 120 μm and process speed of 40 mm/s. A constant laser power of 100 W and a powder layer thickness of 30 μm were used. A relative density of 97.53%, and therefore a low porosity, was obtained at an energy density of 12.5 J/mm^2^. Microscopic examination revealed the presence of small cracks and a very heterogeneous distribution of the grain size.

## 1. Introduction

Tungsten carbide (WC)-based hardmetals are widely used as appropriate material for cutting tools, molds, and mineral applications due to their very good properties in terms of high hardness and elastic modulus, low coefficient of thermal expansion, high wear and corrosion resistance, chemical and thermal stability during high temperature operations [1,2]. The mixing of the hard and brittle WC particles with a ductile binder produces a composite with optimal mechanical properties. Cobalt (Co) is the most common used binder element for WC-based hardmetals and some other metals, such as nickel (Ni) and iron (Fe), are used as alternatives. Ni has been found to reduce wear rate in dry sliding wear experiments because of the softening effect on the microstructure [3]. Moreover, the addition of chromium (Cr), molybdenum (Mo), and/or Ni can significantly improve the resistance of the binder to corrosion [4]. Instead, mechanical properties strongly depend on the microstructure that the manufacturing process gives to the product. In particular, in the WC-based hardmetal sintered parts, the binder phase content, the WC grain size, the mean free path of the binding phase, and contiguity of WC grains are the factors that mainly affect the microstructure [2]. Spherical WC-Co powder has been found to get better results in terms of density thanks to higher apparent density [5]. Moreover, particle size plays an important role in the final quality of sintered products. In fact, nano-particles can improve physical and mechanical properties, but can lead to porosity and structural discontinuities in the sintered parts [6]. WC-based hardmetal components are mainly produced by liquid phase sintering process and solid state processes, such as mechanical alloying. In recent years, non-conventional techniques have been developed with the purpose of reducing the grain growth and to preserve the nanocrystalline structure [2]. Furthermore, WC-Co-Cr thin coatings are often fabricated using the HVOF (high velocity oxy-fuel) process which, if optimal parameters are used, can result in a dense microstructure with 0.6% porosity and good mechanical properties [7].

Selective laser melting (SLM) is, currently, one of the most important additive manufacturing (AM) technologies for producing metal parts. It was developed from the selective laser sintering (SLS) process, which was the first commercialized powder bed fusion process, with the purpose to produce near full density functional parts with high mechanical properties, without the need for post treatments [8,9]. In fact, while the SLS necessarily required the use of post-processing to obtain functional metal parts, with a near full density and mechanical characteristics close to those of the bulk material, in the SLM it is not indispensable, although some post-processes could be used to improve particular features such as fatigue strength and lifetime, distortions and microstructure homogeneity [10,11,12,13,14].

Manufacturing WC carbides using the SLM process could be an alternative method to produce difficult-to-machine and near net shape hardmetal parts. In recent years, several studies have been carried out on SLS and SLM of these materials [1,4,15,16,17,18,19,20,21]. The greatest difficulties encountered in processing WC-based hardmetals through these processes concern the residual porosity and the formation of cracks. Uhlmann et al. [20] stated that the formation of brittle phases, cracks and pores are still not completely avoidable. Full density SLS WC-Co parts could be produced only using a post-processing infiltration treatment [22]. However, even if specimens with a relative density of up to 98% were manufactured, all of them were more or less affected by very deep cracks [21]. As a result, nowadays it is not possible to fabricate functional tools with the required quality [20]. Khmyrov et al. [23] found that only by using a maximum amount of 25 wt% of WC was it possible to fabricate small WC-Co parts without cracks. In fact, they stated that increasing the amount of WC to 50 wt% formation of cracks could not be avoided.

Thus, it is still necessary to carry out extensive research on the printability of WC-based hardmetals by SLM, focusing on process parameters and new powder compositions. The most studied WC-based hardmetals for the manufacture of components by means of SLM have only Co as binder, with percentages higher than 12 wt% [5,21,24]. Since the addition of Cr could increase the abrasion and corrosion resistances of the WC-based hardmetals, especially in severe applications such as the fabrication of metal cutting tools and components for the oil and gas industry, this work focuses on the printability of a WC/Co/Cr powder with a very high WC percentage. The possibility of printing such a material could significantly increase the performance of parts with high value. To date, no scientific works exist focusing on WC/Co/Cr hardmetals processed using SLM. Experiments were conducted using a constant laser power of 100 W and a powder layer thickness of 30 μm. Parallelepiped samples were fabricated and characterized in terms of density, defects, microstructure and hardness.

## 2. Materials and Methods

**Figure 1:**
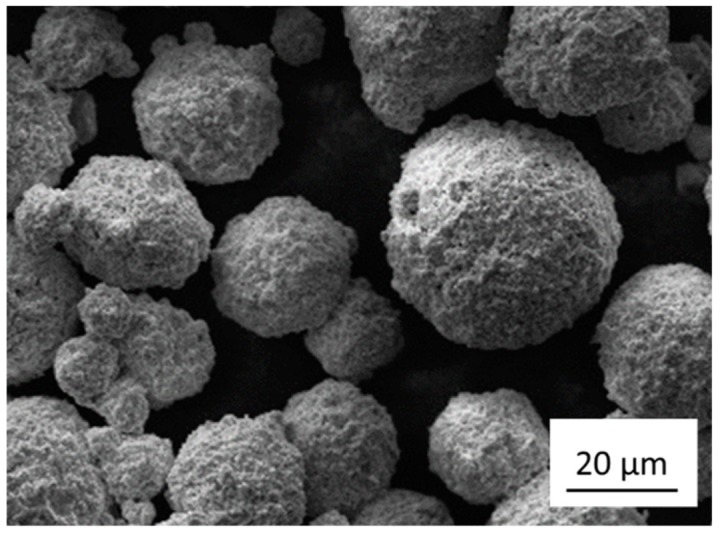
Scanning electron microscope (SEM) image of the powder.

The WC/Co/Cr powder used was the Sintered T311, a commercial spherical powder (Figure 1) produced through gas atomization by MBN Nanomaterialia S.p.A. (Vascon di Carbonera, Italy) [25,26]. This powder is characterized by an elevated amount of WC (about 86 wt%) and by the presence of Co and Cr. The nominal composition of the powder can be found in Table 1. The particle size distribution (PSD) of the T311, as certified by the manufacturer, is shown in Table 2.

Co is the most common binder in WC composites due to high melting point (1493 °C) and its resistance to high temperatures. At 1275 °C it generates a liquid phase with WC and forms an eutectic that melts about 10% of WC. In the subsequent re-solidification, the dissolved carbide re-precipitates conferring resistance to the piece. WC does not dissolve Co, so a small percentage of Co is sufficient to act as binder [4,22,25]. The presence of Cr, in addition to Co, leads to the formation of a binding matrix with more abrasion and corrosion resistances with respect to only Co alloy. For this reason, WC-10Co4Cr alloy is a good alternative to hard chrome surface treatment. Moreover, Cr acts limiting the growth of the WC grains during sintering and causing a significant reduction of the melting temperature of the binder phase [27]. As stated by Frisk and Markström [28], Cr decreases the melting temperature by over 100 °C, thanks to its high solubility in liquid Co.

To test the printability of Sintered T311 powder, a M1 Concept Laser machine, equipped with a 100 W Rofin Nd:YAG laser source, was employed. Three parallelepiped 15 × 15 × 7 mm^3^ multi-layer samples were fabricated with the same process parameters, in order to have statistical significance of results. Laser power (*P*) and layer thickness (*t*) were set to 100 W and 30 μm respectively and a spot diameter (*d*) of 200 μm was used. A random scanning pattern was selected, with a hatch spacing (*Hs*) of 120 μm and a scanning speed (*S*) of 40 mm/s. The energy density, defined as *E*_d_ = *P*/*d*·*S*, was equal to 12.5 J/mm^2^. These values of process parameters were identified through preliminary tests in which samples of only 5 layers were made. The values of process parameters used in the preliminary tests were identified through the study of the available scientific bibliography about the SLM applied to WC-based hardmetals, even if with compositions which differed from that one processed in the present work. In this regard, two important experimental works were those of Khmyrov et al. [18] and Uhlmann et al. [21]; based on these articles and considering that the spot diameter could not be modified (*d* = 200 μm), it was decided to use the maximum available laser power (*P* = 100 W), a hatch spacing equal to the 60% of the spot diameter (*Hs* = 120 μm), a thickness equal to the minimum allowed by the machine (*t* = 30 μm), a scanning speed (*S*) variable on seven levels in the range 40–100 mm/s. In this way, energy density (*E_d_*) values in the range 5–12.5 J/mm^2^ were obtained. For each value of *S,* 3 samples with 5 layers were built. Although all the samples were well anchored to the steel building platform, it was noted that as *S* decreased, and therefore *E_d_* increased, the roughness decreased. Since the aforementioned works also referred to a reduction in porosity, in thicker samples, as the energy input increased, the lowest value of *S* (40 mm/s) was considered the optimum for the following printability tests; in this manner the *E_d_* has been maximized (12.5 J/mm^2^). After building of samples, the construction platform was extracted from the machine and they were removed by means of wire electrical discharge machining (EDM).

Density measurements were carried out by using a KERN ALJ 220-4NM precision balance (Kern & Sohn GmbH, Balingen, Germany), equipped with Archimedes’ method tools. Through this method, the specimens were weighed both in air and subsequently in ethanol after coating with lacquer. The coating was used to avoid the absorption of ethanol in the sample pores during measurement. Metallographic specimens were obtained according to ASTM B665-12 standard procedure [29]. Samples were sectioned using a SiC cut-off wheel and then were hot mounted in phenolic resin. The grinding step was carried out using resin bonded diamond wheels. In the polishing step a 3 µm diamond suspension on a synthetic short-napped cloth was used. Macro- and microstructure were examined using a Nikon Eclipse MA200 inverted optical microscope (Nikon Corporation, Tokyo, Japan). A two-step chemical etching was performed in order to reveal the different phases in the SLM material, as indicated in ASTM B657-18 [30]. Both steps required a Murakami’s reagent, constituted by a freshly prepared mixture of equal quantities of 10 wt% aqueous solution of potassium ferricyanide and sodium hydroxide, used at room temperature. The first step lasted 10 s (revealing η phase) whereas the second step lasted 3 min (revealing α, β and γ phases) [30]. The low-force Vickers hardness tests were performed by means of a Shimadzu HMV-G20ST Vickers hardness tester (Shimadzu Corporation, Kyoto, Japan).

## 3. Results and Discussion

### 3.1. Density

For each of the three fabricated specimens 5 density measurements through the Archimedes’ methodology were carried out, for a total of 15 measurements. An average density of 14.23 g/cm^3^, with a standard deviation of 0.52%, was found. Moreover, the relative density of built samples was calculated considering the density of the bulk material. The density of the bulk material (*D*_WC-Co-Cr_) was calculated as the weighted average of the densities of tungsten carbide (*D*_WC_ = 15.6 g/cm^3^), Co (*D*_Co_ = 8.9 g/cm^3^) and Cr (*D*_Cr_ = 7.14 g/cm^3^) and getting *D*_WC-Co-Cr_ = 14.59 g/cm^3^. Comparing this value with that one obtained by measurements of the built samples, a relative density of 97.53% was obtained. These results are coherent with those found by Uhlmann et al. [21] for a WC-Co material.

### 3.2. Macro- and Microscopic Examinations

**Figure 2:**
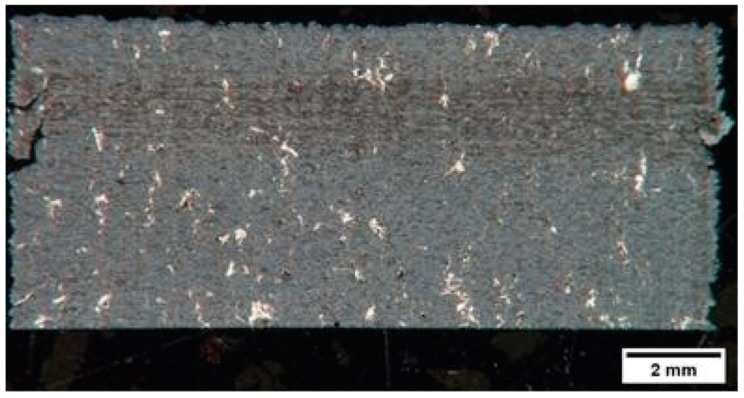
Macrograph of a cross section of a specimen.

**Figure 3:**
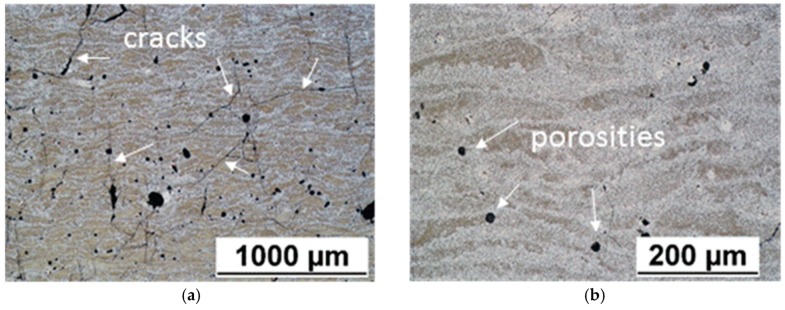
Micrographs revealing (**a**) cracks and (**b**) porosities of a specimen cross section.

Figure 2 shows the macrograph of the sample in the as polished state. The whitish areas represent Co binder accumulation zones due to the used SLM strategy and parameters [27]. From a macroscopic examination it can be deduced that significant macro-defects have not affected the specimens. However, at higher magnification it was possible to see various discontinuities, constituted by an extended net of small cracks (Figure 3a) and porosities with limited size (Figure 3b). The limited size of porosity is related to the relative high density achieved.

As already mentioned in the introduction section, the formation of cracks was expected. In fact, in the literature it has been demonstrated that a high amount of hard phases is responsible for the formation of cracks in WC-Co composite materials built by SLM [23]. However, not macro-cracks were obtained, but only micro-cracks. This is a significant result. Behind the cracks formation there are many phenomena, as has already been discussed in the literature. Basically, cracks are due to the high solidification rate of molten pool, to the high melting point difference and thermal expansion coefficient mismatch between WC and binder materials, to the precipitation of brittle phases, and to the ductile-to-brittle transition that, in tungsten, occurs above room temperature [21,23,24,31,32].

In the printability test presented, the material was subjected to a thermal load of about 2.5 × 10^5^ W/cm^2^, that can be one to two orders of magnitude higher than the thermal loads in foundry applications [32]. Because of the very localized heat supply, the material suffered a considerable thermal shock. The extremely high temperature difference between the molten pool and the rest of the part is the main cause of relevant thermal stresses, that after cooling to room temperature, determine the presence of very high longitudinal and transversal tensile residual stresses in SLM tracks [33]. These residual stresses in combination with the ductile-to-brittle transition of tungsten, which falls on average between 200 °C and 400 °C, and with the thermal expansion coefficient mismatch between WC and binder materials, can be a cause of the observed cracks [32]. Moreover, given the high melting point difference between the binder and the WC, the evaporation of the binder is likely to have occurred, which would have caused an unwanted change in the initial chemical material composition, reducing its toughness and resistance to fracture, as also has been reported in the literature [20]. According to previous experimental works on SLM of brittle materials, some effective methods to try to reduce or eliminate cracking could be the optimization of the scanning strategies, the reduction of energy density, allowing a reduction of maximum temperature and binder losses, and the preheating of the building platform, to limit the thermal shocks [21,23,32].

Critical issues related to the initial chemical composition of the powders and the binder losses are extensively discussed in literature for different materials. Maamoun et al. [34] showed the role of Si content in microstructure evolution and hot cracking reduction in Al-alloys processed by SLM. Li et al. [35] used a NiAlCoCrCuFe alloy as binder element to reduce the Co content, promoting solid solutions and oxidation resistance.

**Figure 4:**
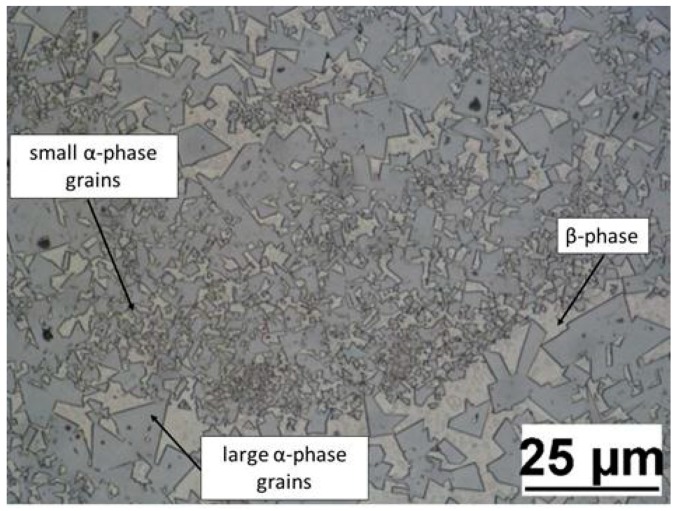
Micrograph of a sample cross section at 1000× magnification with the identification of α and β phases.

Figure 4 shows an optical micrograph of the sample cross section in the as polished state. In this micrograph a microstructure consisting of the α-phase (facetted WC grains) embedded in the β-phase (Co rich binder) can be observed. WC grains are not completely surrounded by the binder, but they are often in contact with other WC grains and the shape of the grains is typically polygonal. The grain size has a significant heterogeneous distribution due to the layer-by-layer laser melting/solidification process. In fact, due to the very high cooling gradient, smaller WC grains are generated in the molten beads and larger grains along their borders. Moreover, each layer, after the solidification, was subjected to a reheating cycle during the melting of the successive layer. The reheating cycle affected only the upper zones of each layer, which were adjacent to the new melting area. This phenomenon lead to the coarsening of only some of the WC grains of each layer. Globally, grains appeared cyclically fine and coarse until the SLM process finished. Unfortunately, this microstructural configuration caused a remarkable embrittlement of the material [21].

**Figure 5:**
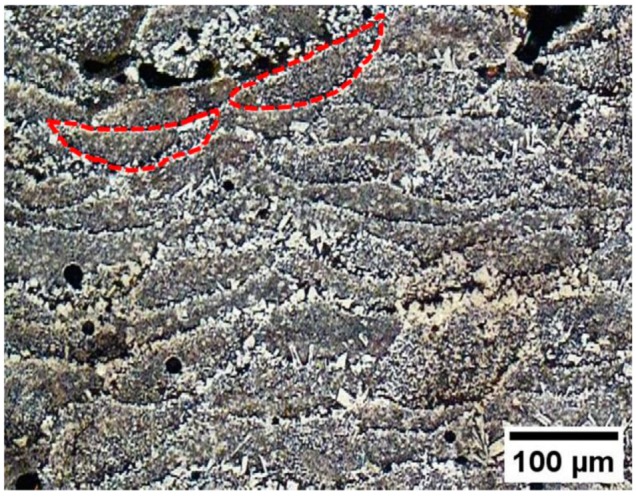
Micrograph of a sample cross section revealing the molten pool shape.

Figure 5 shows a micrograph of a sample cross section after a light etching with the Murakami’s reagent. This micrograph reveals the molten pool shape, which appears to be quite variable due to the selected random scanning pattern: with this strategy, the scanning direction changed moving from one layer to another. However, the molten pool shapes of two tracks sectioned perpendicularly are dashed in red: they are very similar.

**Figure 6:**
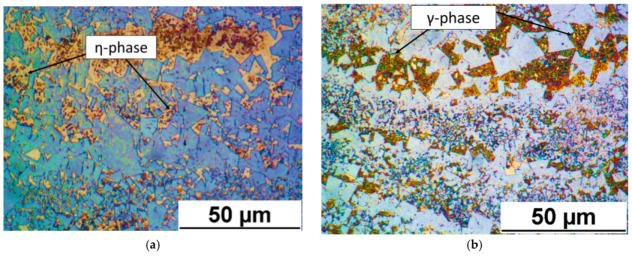
Micrographs revealing (**a**) η-phase (round shaped and blue coloured) and (**b**) γ-phase (yellow to brown coloured with round shape).

Figure 6a,b show the microstructure of the samples. In Figure 6a it is possible to distinguish the η-phase (round shaped and blue coloured) representing (Co,Cr)_3_W_3_C constituent [30]. In order to determine the presence of γ-phase, a second etching with the same reagent, but for a longer time was applied on the same cross section. In Figure 6b γ-phase (yellow to brown coloured with round shape) is visible [30].

An important microstructural parameter for this type of material is the contiguity *C* of WC particles. Contiguity is defined as “the fraction of the total internal surface area of a phase that is shared by particles of the same phase” [36]. The evaluation of *C* was performed because it is related to the hardness and brittleness of the material [37]. Lee and Gurland’s formula of *C* is the following [36,37]:

(1) $$C=1-\frac{V_{b}}{\left(1-V_{b}\right)\cdot \frac{L_{b}}{L_{WC}}}$$

where *V_b_* is binder volume fraction, *L_WC_* [µm] is WC grain size and *L_b_* [µm] is the binder mean free path. *V_b_* was calculated by the sum of the Co and Cr volume fractions and was equal to 0.234.

*L_WC_* distribution was evaluated according to ISO 4499-1:2008 by the image comparison method [27,38]. The results of the evaluation showed that *L_WC_* fell within four classes, from “fine” to “extra coarse”. Another evaluation of the grain size distribution was performed according to ISO 4499-2:2008 [39]. The intercept method was used onto four fields with 90 × 90 µm^2^ area for each of the three fabricated samples. Table 3 summarizes the obtained mean values of WC grain size distribution for the three examined samples. The mean and the maximum *L_WC_* measured in the examined fields are 2.1 µm and 12.9 µm respectively.

*L*_b_ represents the average linear distance between WC contiguous grains [38]. This parameter can be easily estimated in image analysis by measuring the length of linear intercepts of the binder phase with a set of parallel lines in a cross section. Results of the mean free path *L*_b_ evaluation are reported in Table 4. The mean and the maximum *L*_b_ in the examined cross sections are 2.4 µm and 16.5 µm respectively.

Considering mean values of *L*_WC_ and *L*_b_, Equation (1) gives a value of 0.733 for *C*. It is possible to compare this result with those reported in Luyckx and Love [36], even if in the latter the binder was constituted only by Co: at the same binder volume fraction, samples realized in this work had a value of *C* much higher than those mentioned by Luyckx and Love. In fact, Luyckx and Love found a value of C ranging between 0.25 and 0.37. As already stated by Lee and Gurland [37], the values of contiguity also depends on the manufacturing process; Luyckx and Love investigated the contiguity of the carbide phase of parts made by traditional sintering, while in the present work samples obtained by SLM were analysed. The high value of *C* indicates the presence of a continuous carbide skeleton in the built sample, but, in contrast, a highly concentrated stress at the carbide-carbide contact interfaces is expected, leading to the formation of a net of small cracks, as shown in Figure 3a.

### 3.3. Hardness

Low-force Vickers hardness tests were performed on the cross sections of the three specimens. Tests were carried out applying, in 5 different positions for each cross section, a load of 1 kg (HV1). The results are listed in Table 5.

**Figure 7:**
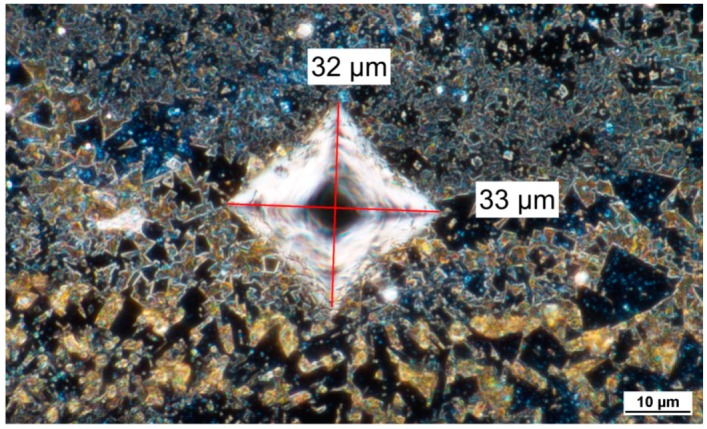
Micrograph of a Vickers hardness test indentation.

The measured hardness values are in agreement with the results found by Nanda Kumar and Kurokawa [40]. They found that temperatures and thermal gradients typical of the fabrication method are responsible of changes in chemical composition at grain boundaries and of the high variability in grain size. Furthermore, as explained in Section 3.2, the grain size had a heterogeneous distribution due to the layer-by-layer laser melting/solidification process. For these reasons, the range of variability of hardness (about 298 HV1), shown in Table 5, may seem very high if compared to absolute values, but it is consistent with those found in the literature [1,40]. Finally, the micrograph in Figure 7 proves the variability of both the WC grains size and the amount of binder, characterizing the area of each hardness indentation. In fact, in the upper part of indentation it is possible to observe an area with a prevalence of small α-phase grains, in the lower part an area with a prevalence of large α-phase grains and in the central part an area with a prevalence of β-phase, and therefore of binder.

## 4. Conclusions

This experimental work focused on the printability of a WC/Co/Cr powder with a very high WC percentage (86 wt%) by means of SLM technology. The most studied WC-based hardmetals for the manufacture of components by means of SLM had only Co as binder, but it is known that the addition of Cr could increase the abrasion and corrosion resistances of the WC-based hardmetals, especially in severe applications such as the fabrication of metal cutting tools and components for the oil and gas industry. Therefore, the possibility of printing a WC-10Co4Cr material could significantly increase the performance of parts with high value. However, before carrying out extensive research on the SLM fabrication of such a material, it was worth proceeding to a test of the printability of it. In fact, to date, no scientific works exist on WC/Co/Cr hardmetals processed using SLM. The values of the set of process parameters tested were identified through the study of the available scientific research on SLM applied to different WC-based hardmetals, and through preliminary tests in which samples of only 5 layers were fabricated. The main remarks are listed in the following.

- At an energy density of 12.5 J/cm^2^, corresponding to a laser power of 100 W and a scan speed of 40 mm/s, a relative density of 97.53%, and therefore a low porosity, was obtained.
- No macro-cracks but only a net, although extended, of micro-cracks affected the SLM samples. This is a substantial result, given the high fragility of the processed material and the remarkable tensile residual stresses generated by the SLM process.
- The presence of a net of small cracks was also confirmed by the calculation of contiguity of WC particles, which was equal to 0.733, a very high value depending both on the low binder volume fraction of the used WC/Co/Cr powder and on the SLM process.
- Microscopic examination revealed that grain size had a very heterogeneous distribution due to the layer-by-layer laser melting/solidification process. WC grain size fell within the classes from “fine”to “extra coarse”; the mean and the maximum measured WC grain size were 2.1 µm and 12.9 µm respectively.
- The mean value of Vickers hardness was 2001 HV1 and its range of variability of 298 HV1 was very high, depending itself on the variability both of WC grains size and of the amount of binder characterizing the area of indentation.

The results of this work encourage a deepening of experimentation, considering that some effective methods to attempt to reduce or eliminate cracking could rest on optimization of scanning strategies, the reduction of energy density, allowing a reduction of maximum temperature and binder losses, and the preheating of the building platform, to limit the thermal shocks. The next experimental step will involve the development of a proper experimental plan in order to optimize the manufacturing of SLM parts in WC-10Co4Cr material.

## Figures and Tables

**Table 1:** Chemical composition (wt%) of the powder.

Type of Powder	Co	Cr	WC
Sintered T311	10.0	4.0	86.0

**Table 2:** Particle size distribution (PSD) of the powder.

PSD	Particle Size [μm]
D10	16
D50	35
D90	49

**Table 3:** Results of the *L_WC_* evaluation by intercept method.

*L*_WC_ Classes	<1 µm	1–5 µm	>5 µm	Total
**Number of Grains**	70	129	17	216
**Standard Deviation**	18.4	2.0	2.6	18.2
**Relative Quantity [%]**	32.4	59.7	7.9	100

**Table 4:** Results of the mean free path *L_b_* evaluation by intercept method.

*L_b_* Classes	<1 µm	1–2 µm	2–5 µm	>5 µm	Total
**Number of Intercepts**	55	121	75	22	273
**Standard Deviation**	7.2	15.5	7.8	3.0	11.1
**Relative Quantity [%]**	20.1	44.3	27.5	8.1	100

**Table 5:** Vickers hardness results.

	Min	Max	Mean Value	Range of Variability
**Vickers hardness [HV1]**	1840	2138	2001	298
